# Fifty Shades of Unsaid: Women’s Explicit and Implicit Attitudes Towards Sexual Morality

**DOI:** 10.5964/ejop.v12i4.1124

**Published:** 2016-11-18

**Authors:** Tiziana Lanciano, Emanuela Soleti, Francesca Guglielmi, Ivan Mangiulli, Antonietta Curci

**Affiliations:** a Department of Education, Psychology, Communication, University of Bari “A. Moro”, Bari, Italy; University of Neuchâtel, Neuchâtel, Switzerland

**Keywords:** sexual morality, implicit attitudes, erotophobia-erotophilia, sex guilt

## Abstract

The movie Fifty Shades of Grey has created a great deal of controversy which has reignited the debate on unusual and alternative sexual practices such as bondage. Erotophobic individuals have negative affect towards the type of sexual libertinism conveyed by the movie, while erotophilic persons have a positive attitude and emotional feelings towards this kind of sexual emancipation. Using the Implicit Association Test, this study aimed to explore the extent to which there is a difference in women's attitudes towards sexual morality on an explicit and implicit level. Our findings found that erotophobic and erotophilic women differed only on an explicit level of sex guilt and moral evaluation, while no difference in the implicit measure was found.

## Introduction

There can be no doubt that the movie adaptation of the book Fifty Shades of Grey (De Luca, Brunetti, James, & Taylor-Johnson, 2015) has been a phenomenon. There has been no more eagerly anticipated movie this year (Robey, 2015). In 2011, British author E. L. James’s novel (the first of the trilogy) was published by Vintage Books, while Hollywood released a movie adaptation of the book on Valentine’s Day 2015. Both the trilogy and the movie depict a romantic and erotic Bondage-Domination-Sadism-Masochism (Cooper, 2012; Weiss, 2011) relationship concerning 28-year-old millionaire Christian Grey and 22-year-old college graduate Anastasia Steele (James, 2011). To illustrate, Bondage-Domination-Sadism-Masochism refers to a range of behaviors in a sexual context characterized by fetishes, role-playing, and nonmainstream activities, which includes aspects of power and pain and where there is the consent of all parties involved (Barker, Iantaffi, & Gupta, 2007; Weiss, 2011). Although there is debate about what may be considered “acceptable” within the context of Bondage-Domination-Sadism-Masochism, many practices are painful and go on to propagate traditional gender role dynamics, such as aggressive performances (Barker, Iantaffi, & Gupta, 2007). In Fifty Shades of Grey, the couple’s Bondage-Domination-Sadism-Masochism practice involves Christian as the dominant and Anastasia as the submissive partner. One the most perverse aspects of the story is Anastasia’s agreement to sign a dominant/submissive contract with Christian (James, 2011).

Astonishingly, the book has sold over 100 million copies and the movie has also been a worldwide success. The phenomenon of the book and movie is energized by their appeal to modern women, who manage both a family and their career (Schneiderman, 2012). These twenty-first century modern women have bought the book in record numbers. According to radical-libertarian feminists, women should retract any moral judgements that stigmatize sexual practices and constrain their sexual freedom. Women should claim the right to experience anything which gives them pleasure, gratification, and/or satisfaction, and the right to experiment with a variety of sex practices without limiting themselves to a restricted range of sexual experiences (Ferguson, 1984; Tong, 2013). In other words, Fifty Shades of Grey has been considered as female romance porn for the hookup generation in both narrative and movie forms (Eckman, 2015; Schneiderman, 2012).

### Sexual Morality vs. Sexual Emancipation

Generally, the world of sex and sexuality involves a massive variety of emotions and personal values and connotations. For example, sex may include both the negative emotions of moral judgments and painful recollections of earlier sexual experiences, and positive feelings, fantasies, desires and pleasant reminiscences (Macapagal & Janssen, 2011; Peterson & Janssen, 2007). The dimension of personality broadly used to assess sex-related emotions and attitude is the erotophobia-erotophilia dimension (Fisher, Byrne, White, & Kelley, 1988). The bipolar single trait of erotophobia-erotophilia assesses the individual’s variability to openness-closeness to sex and sexuality, which are linked to people’s approach-avoidance inclinations in sexual situations. To illustrate, erotophobic people have very negative attitudes to sex; they feel a wide range of emotions towards sexuality, including anger, vulnerability, disgust, fear, and shame. Literature suggests that erotophobic individuals tend to have higher levels of totalitarianism and need for success, have more traditional sex roles (Fisher, Byrne, et al., 1988) and lower sexual familiarity and are less likely to ask for sex education than erotophilic people (Fisher, Grenier, et al., 1988; Gerrard, Kurylo, & Reis, 1991). Consequently, erotophobic people tend to avoid any sexual context, above all when the risk of a sexual intercourse is high and unexpected.

On the contrary, erotophilic people have a positive attitude and emotional feelings towards sex; they consider sex as a positive part of life (indeed "sex positive" is used as a term along with erotophilia). An erotophilic person embraces all facets of sexuality, including unusual practices such as bondage, dominance and submission. Erotophilic individuals think about sex more often, tend to masturbate and fantasize more frequently, have their first sexual intercourse at an earlier age and have more intercourse partners than erotophobic individuals. Erotophilic people are also more likely to engage in regular gynecological visits and in preventative behaviors concerning sexually transmitted diseases (Fisher, Byrne, et al., 1988), and are more inclined to debate sexual care, than erotophobic individuals (Herbenick, Reece, & Hollub, 2009).

In this vein, Fifty Shades of Grey has created controversy in readers and viewers both on the internet and newspapers, leading people to rekindle the debate on sexual matters and unusual practices. From the first days of the movie’s release, blogs and social networks were full of opinions about its core concepts from both opponents and fans alike. The Fifty Shades trilogy and movie are a product of a modern civilization and a twenty-first century culture that encourages people to be sexually free and independent and to take pleasure in anyone and anything they desire without guilt (Douthat, 2015). According to Catholic communities, the irrationality of Fifty Shades of Grey is that Mr. Grey is a man who first dominates Miss Steele but finally loves her, providing that she sign a challenging contract containing all types of domination that she will take part in (Eckman, 2015). We live in a society with a bland sense of sex-related shame, and sex corresponds to an attack on human self-respect and dignity (Mohler, 2015). While progressive culture considers Fifty Shades as cultural progress, for some people it is sign of cultural worsening and failure (Eckman, 2015).

Regardless of the sexual context conveyed by the movie, what has been said underlines the idea that sex may elicit mixed affective and cognitive responses and evaluations such as positive affect, pleasantness, satisfaction, a sense of freedom, but also fear of pleasure, shame, or guilt. Sex guilt has been defined as “a generalized expectancy for self-mediated punishment for violating or for anticipating violating standards of proper sexual conduct. Such a disposition might be manifested by resistance to sexual temptation, by inhibited sexual behavior, or by the disruption of cognitive processes in sex-related situations” (Mosher, 1998, p. 27).

Erotophobic individuals feel guilt for partaking in particular sexual activities such as Bondage-Domination-Sadism-Masochism, oral sex, masturbation, fantasies about sex and same-sex partners, and judge these practices as immoral, sinful, unclean and dirty. Sex guilt is generally associated with the feeling of being dirty. Who experience sex guilt usually think that sex is degrading and connected to primitive and animal instincts, and they approach sex as an manifestation of lack of self-control (Sinclair Intimacy Institute, 2015).

### Explicit and Implicit Attitudes Towards Sexuality

Many studies on affect and sexuality have focused on the conscious processes of behaviors and reactions to sexual stimuli, and have generally investigated individuals’ immediate and consciously experienced reactions to sexual stimuli (Wiegel, Scepkowski, & Barlow, 2007). Nevertheless, a few studies have investigated the possibility that sex-related affective attitudes may involve automatic processes which occur outside an individual's conscious awareness and are engaged immediately and without conscious effort when sexual stimuli are given (Brauer, de Jong, Huijding, Laan, & ter Kuile, 2009; Oliver, Watson, Gannon, & Beech, 2009; Macapagal & Janssen, 2011).This is indeed the open issue which drives our pilot study: Do women who explicitly evaluate themselves as sexually liberal, emancipated and revolutionary, implicitly conceptualize sexual perversions (such as Bondage-Domination-Sadism-Masochism practices) as not being morally guilty and dirty? We suppose that attitudes towards Bondage-Domination-Sadism-Masochism practices may be vulnerable to the bias of explicit self-report measures (i.e. questionnaires), and that implicit measures may be more suitable to detect these attitudes.

In the present pilot study we examined individuals’ representations concerning an emancipated and liberal sexuality, as that conveyed by the movie Fifty Shades of Grey, by comparing women’s implicit and explicit attitudes. Among implicit measures, the Implicit Association Test (Greenwald, McGhee, & Schwartz, 1998; Greenwald, Nosek, & Banaji, 2003) was considered as providing a crucial step forward. Generally, the label implicit refers to assessment techniques that control the role of awareness in producing response and self-reflective processes (Nosek, Greenwald, & Banaji, 2007). Greenwald and colleagues claim that individuals are not always conscious of their social attitudes (Greenwald et al., 1998), and because of this, the IAT is built to explore attitudes which participants are unaware of or reluctant to express for fear of being judged as having politically incorrect opinions. It is a method used to indirectly assess the power of relations among concepts, and requires the categorization of stimulus exemplars from four concepts choosing just two response options, each of which is assigned to two of the four concepts. The idea underlying the IAT is that this sorting task should be easier and faster when the two concepts that share a response are strongly associated than when they are weakly associated (Nosek et al., 2007).

A large body of research has provided findings concerning implicit attitudes towards homosexuality (Anselmi, Vianello, Voci, & Robusto, 2013; Banse, Seise, & Zerbes, 2001; Breen & Karpinski, 2013; Jellison, McConnell, & Gabriel, 2004; Jones & Devos, 2014; Steffens, 2005). Other studies did not directly investigate sex-related implicit attitudes, but instead used the concepts of implicit and explicit processes in sexuality, such as explicit vs. implicit memory for sexual words (Bush & Geer, 2001), implicit vs. explicit priming of the sexual system (Spiering, Everaerd, & Janssen, 2003), or implicit attitudes toward mainstream sexual terms (e.g., kissing) and Bondage-Domination-Sadism-Masochism terms (e.g., bondage) (Stockwell, Walker, & Eshleman, 2010). In a recent study, Macapagal and Janssen (2011) tested the link between automatic associations with sexual stimuli and the dimension of erotophobia-erotophilia. The authors concluded that the valence of sexual stimuli can be treated automatically and this is related to trait affective responses to sex.

Jointly considered, findings showed that sexually implicit stimuli presented outside of awareness had a different impact when compared with explicit stimulus presentations. These findings authorize us to consider both an explicit and implicit level in our investigation of sex-related attitudes.

### Overview and Hypotheses

The present pilot study aimed to explore women’s explicit and implicit attitudes towards an emancipated and liberal sexuality, such as the Bondage-Domination-Sadism-Masochism practice conveyed by the movie Fifty Shades of Grey. Any explanation of the aim of the current study must begin with a crucial consideration: We were not interested in recruiting a sample of movie supporters vs. opponents, also because real opponents of the movie surely did not see it. We aimed instead to investigate the different attitudes (explicit and/or implicit) of people who saw the movie. Our idea is that erotophobic and/or moralist individuals would generally oppose and disapprove of the sexual libertinism shown, and if some of these decided to see the movie, they did so because they consider it to be largely a romantic and loving film. Instead, erotophilic and/or emancipated individuals may support and encourage the sexual libertinism displayed in the movie.

We aimed to explore the extent to which there is a difference in attitudes towards sexual morality on an explicit and implicit level. To test our aim, we randomly selected women older than 30 years of age who had seen the movie, and their dispositional traits of erotophobia-erotophilia were assessed. The choice of a totally female sample is due to the movie’s prospective of female submission in which the Bondage-Domination-Sadism-Masochism practices involve the male protagonist as the dominant partner and the female protagonist as the submissive partner. Empirical evidence suggests that about 89% of women who are engaged in Bondage-Domination-Sadism-Masochism expressed a preference for a dominant man (Ernulf & Innala, 1995). Women were also administered a sexual satisfaction measure in order to exclude attenuate confounding due to sexual satisfaction or sexual distress. Additionally, we operationalized the concept of an emancipated and liberal sexuality through some Bondage-Domination-Sadism-Masochism photograms taken from the Fifty Shades of Grey movie. We also operationalized the concepts of sexual morality/immorality in terms of cleanliness/dirtiness. Indeed, the main strength of the current study is that it does not adopt the traditional IAT “good/bad” attribute contrast (Greenwald et al., 2003), but instead adopts the categories of "dirty" and "clean". Empirical evidence has shown that abstract concepts of morality/immorality are grounded in situations of physical cleanliness/dirtiness, in that water and soap eliminate more than physical dirt and weaken guilt from an individual’s moral misbehaviours (Lakoff & Johnson, 1999; Lee & Schwarz, 2010; Schnall, Benton, & Harvey, 2008; Zhong & Liljenquist, 2006). Indeed, we do not wish to investigate the conceptualization of sex in terms of goodness/badness and consequently we are not interested in the sexual pleasure or satisfaction which we hypothesized as being unassociated with the moralist attitude. We are instead interested in the conceptualization of unusual sex practices in terms of morally dirty and guilty attributes. Despite the exploratory nature of the current study:

H1: At the explicit level, we expected erotophobic women to exhibit higher levels of sex guilt, moral and romantic evaluation of the movie, and to declare that their motivation for seeing the movie was connected to the media phenomenon surrounding the film or that they had seen the movie by mistake. (See Measures section for a detailed explanation).

H2: At the explicit level, we expected erotophilic women to exhibit higher levels of fantasies and reflections after seeing the movie, a higher sexual evaluation of the film, and to give sexual motivations as the underlying reason for having seen the movie.

H3: At the implicit level, we expected all participants (both erotophobic and erotophilic women) to show a high IAT effect. We also expected no difference between the two groups in IAT effect to be found. In other words, along with erotophobic women, we also expected erotophilic women who explicitly assessed themselves as being sexually emancipated, libertine and modern, to associate the sexual perversions portrayed in the movie with the concept of dirt.

H4: We expected that explicit and implicit measures would not converge to discriminate women’s attitudes towards sexual morality. We also expected that the IAT would not be able to detect the differences between erotophobic and erotophilic attitudes found at the explicit level.

## Material and Methods

### Design

The study was approved by the Ethical Committee of the Department of Education, Psychology, Communication of University of Bari. The study adopted a 2x2 mixed design with the IAT block (congruent vs. incongruent) as within subjects, and the explicit sex attitude (erotophobic vs. erotophilic) as between subjects. The dependent variables were: (1) explicit measures of sexual satisfaction, sex-guilt, affective responses to the movie, evaluation of the movie, motivations for viewing the movie, and (2) the implicit measure of the IAT effect (Greenwald et al., 1998).

### Participants

A sample of 25 female Italian volunteers (age range = 31-64; *M* = 46.88; *SD* = 7.20) was recruited through an advertisement and among the researchers’ acquaintances through a snowball sampling method (Biernacki & Waldorf, 1981). To distinguish the two explicit sex attitude groups (erotophobic vs. erotophilic), a median-split procedure was applied to the measure of sexual attitude (Macapagal & Janssen, 2011; see Measures section). On the basis of the median score (score = 70), we distinguished erotophobic vs. erotophilic women.

The two groups were homogenous as regards age (*t*(23) = 1.33, *p* = .20), educational level (χ^2^(2, *N* = 24) = 1.48, *p* = .48), length of relationship (*t*(21)= 1.19, *p* = .25), marital status (χ^2^(2, *N* = 24) = 2.73, *p* = .26), motherhood (χ^2^(1, *N* = 25) = 0.36, *p* = .55), number of children (χ^2^(3, *N* = 25) = 1.65, *p* = .65), date of viewing before the experiment (χ^2^(2, *N* = 25) = 0.96, *p* = .62), number of times the movie was seen (χ^2^(2, *N* = 25) = 2.36, *p* = .31), other people present when participants saw the movie (χ^2^(4, *N* = 25) = 2.36, *p* = .67), show time (χ^2^(2, *N* = 24) = 5.87, *p* = .53), and previous reading of the trilogy (χ^2^(2, *N* = 25) = 1.13, *p* = .57). Detailed characteristics for the two subsamples are shown in Table 1.

**Table 1:** Characteristics for the Two Subsamples

Characteristic	Erotophobic (*n* = 13)^a^	Erotophilic (*n* = 12)^a^
Age	*M* = 48.69, *SD* = 6.56	*M* = 44.92, *SD* = 7.61
Education		
Middle school	*n* = 0	*n* = 1
High school	*n* = 6	*n* = 4
Degree	*n* = 6	*n* = 7
Length of Relationship	*M*_months_ = 20.19, *SD* = 7.45	*M*_months_ = 15.26, *SD* = 11.73
Marital status		
Single	*n* = 2	*n* = 3
Cohabiting	*n* = 0	*n* = 2
Married	*n* = 10	*n* = 7
Motherhood		
Yes	*n* = 11	*n* = 9
No	*n* = 2	*n* = 3
Number of children		
No child	*n = 2*	*n = 3*
1 child	*n* = 2	*n* = 3
2 children	*n* = 8	*n* = 6
4 children	*n* = 1	*n* = 0
Date of viewing		
Some days ago	*n* = 1	*n* = 1
One week ago	*n* = 1	*n* = 0
More than one week ago	*n* = 11	*n* = 11
Number of times movie was seen		
Once	*n* = 13	*n* = 10
Twice	*n* = 0	*n* = 1
More than 2 times	*n* = 0	*n* = 1
Others		
Alone	*n* = 1	*n* = 2
Partner	*n* = 5	*n* = 2
Sister	*n* = 1	*n* = 2
Friend	*n* = 4	*n* = 5
Other	*n* = 2	*n* = 1
Show time		
Early afternoon	*n* = 0	*n* = 4
Late afternoon	*n* = 3	*n* = 1
Evening	*n* = 10	*n* = 6
Trilogy reading		
No	*n* = 6	*n* = 7
Yes, before movie	*n* = 6	*n* = 5
Yes, partly	*n* = 1	*n* = 0

^a^The cell count may not match the sample size since the missing values.

### Measures and Procedure

#### Explicit Measures

##### Sexual attitude

The participants’ sexual attitudes were assessed through the Sexual Opinion Survey (SOS) (Fisher, Byrne, et al., 1988). The SOS consists of twenty-one 7-point items (1 = “strongly agree”; 7 = “strongly disagree”) which measure normal variations in affective- evaluative responses to sex along a single dimension of erotophobia–erotophilia. Items address participants’ attitudes towards a number of sexual behaviors (e.g., masturbation, fantasizing about sex, same-sex partners, oral sex). Specifically, the scale consists of 10 erotophobia items assessing negative affective responses to sex (e.g., “I do not enjoy daydreaming about sexual matters”; Cronbach’s alpha = .78), and 11 erotophilia items assessing positive affective responses to sex (e.g., “Seeing a pornographic movie would be sexually arousing to me”; Cronbach’s alpha = .91). Participants’ scores on the SOS are obtained by subtracting the sum of the erotophobia items from the sum of the erotophilia items, and a constant of 67 is added to the difference. Higher scores indicated higher erotophilia, while lower scores indicated higher erotophobia.

##### Sexual satisfaction

Participants filled in the Sexual Satisfaction Scale for Women (SSS-W) (Meston & Trapnell, 2005). The SSS-W consists of thirty 5-point items assessing sexual satisfaction and sexual distress (1 = “strongly agree”; 5 = “strongly disagree”). Item scores were summed up into a total score (Cronbach’s alpha = .91), and five sub dimensions: a) contentment (e.g. “I feel content with the way my present sex life is”; Cronbach’s alpha = .76), b) communication (e.g. “I usually feel completely comfortable discussing sex whenever my partner wants to”; Cronbach’s alpha = .72), c) compatibility (e.g. “I often feel that my partner and I are not sexually compatible enough”; Cronbach’s alpha = .93), d) relational concern (e.g. “I’m worried that my partner will become frustrated with my sexual difficulties”; Cronbach’s alpha = .90), and e) personal concern (e.g. “My sexual difficulties are frustrating to me”; Cronbach’s alpha = .96).

##### Sex guilt

Women’s sex guilt was assessed through the brief version of the revised Mosher Sex-Guilt Scale (BRMSGS) (Janda & Bazemore, 2011). This scale represents a brief 10-item version of the 50-item Sex-Guilt Scale from the Revised Mosher Guilt Inventory (Mosher, 1998). The scale consists of ten 7-point items (0 = “totally false”; 6 = “totally true”; e.g., “Unusual sex practices are dangerous to one’s health and mental”). Item scores were summed up into the total score of sex guilt (Cronbach’s alpha = .69). Higher scores indicated higher sex guilt.

Considering that erotophobic women saw the Fifty Shades of Grey movie too, we considered it crucial to explore the affective responses, evaluations, and motivations underlying their viewing of the movie. To address this aim, we needed to create the following specific items.

##### Affective responses to the movie

Participants were asked to assess through sixteen 11-point items (0 = “not at all”; 10 = very much”) the extent to which they felt a) pleasure, b) emotion, c) excitement, d) fear, e) disappointment, f) curiosity, g) sadness, h) indifference, i) erotic fantasies, l) dreams, m) desire for a similar situation, n) reflections on one’s own romantic relationships, o) reflections on one’s own sexual relations, p) shame, q) guilt, and r) dirtiness. Scores of items a), b), c), and f) were averaged into the sub dimension of positive emotions (e.g. “The movie excited me”; Cronbach’s alpha = .87). The scores of items d), e), g), and h) were averaged into the sub dimension of negative emotions (e.g. “The movie disappointed me”; Cronbach’s alpha = .43). The scores of items i), l), m), n) and o) were averaged into the sub dimension of fantasies & reflections (e.g. “The movie prompted erotic fantasies in me” or “The movie made me reflect on my romantic relationship”; Cronbach’s alpha = .82). The scores of items p), q), and r) were averaged into the sub dimension of moral evaluation (e.g. “I felt guilty for having seen the movie” or “I felt dirty for having seen the movie”; Cronbach’s alpha = .95).

##### Evaluation of the movie

Participants were asked to assess through six 11-point items (0 = “not at all”; 10 = very much”) the extent to which the movie was a) erotic, b) hard, c) porn, d) sadomasochistic, e) perverted, and f) loving. The scores of items a), b), c), d), and e) were averaged into the sub dimension of sexual evaluation (Cronbach’s alpha = .79). The score of item f) was considered as an index of romantic evaluation of the movie.

##### Motivations underlying viewing of the movie

Participants were asked to assess through seven 11-point items (0 = “not at all”; 10 = very much”) if they saw the movie: a) to see an erotic film, b) to see hard scenes, c) to get excited, d) to criticize it, e) to comment on it, f) because the movie has been highly publicized, g) by mistake (i.e. no other movie was available). The scores of items a), b), c) were averaged into the sub dimension of sexual motivation (Cronbach’s alpha = .87). The scores of items d), e), f) were averaged into the sub dimension of media motivation (Cronbach’s alpha = .85). The score of item g) was considered as an index of viewing the movie by mistake.

#### Implicit Measures

##### IAT measures

To construct the IAT task, for the Movie vs. No Movie categories we selected eight pictures taken from the Fifty Shades of Grey movie representing BDSM practices, and eight mainstream pictures of weddings, not presented in the movie. For the Dirty vs. Clean categories, we selected eight words belonging to the dirty category and eight words belonging to the clean category (see Table 2 for details). The IAT consisted of five separate blocks of categorization trials (see Table 2). In each trial, a stimulus item (picture or word) was presented in the center of a computer screen, and participants were told to categorise it as accurately and as quickly as possible.

- In Block 1 (Movie categorisation) participants classified each picture as belonging to the movie (press “E”) or not belonging to the movie (press “I”).
- In Block 2 (Moral categorisation) participants classified each word as dirty (press “E”) or clean (press “I”).
- In Block 3 (Double categorisation congruent block) participants evaluated whether stimulus (picture or word) were either belonging to the movie or dirty (press “E”), and whether they were either not belonging to the movie or clean (press “I”).
- In Block 4 (Reversed movie categorization) participants classified pictures as not belonging to the movie (press “E”) or belonging to the movie (press “I”).
- In Block 5 (Reversed double categorization incongruent block) participants evaluated whether stimulus (picture or word) were either not belonging to the movie or dirty (press “E”), and whether they were either belonging to the movie or clean (press “I”).

Error feedback in the form of a red letter “X” appeared after incorrect responses and participants had to correct error responses hitting the other key (built-in error correction procedure). Each stimulus (picture or words) was presented a number of different times within each block, and the order of presentation and number of repetitions of each trial was completely randomized for each participant within blocks. Associations of Dirty vs. Clean words with movie vs. not movie pictures were randomly presented in Blocks 3 and 5.

**Table 2:** Summary IAT Blocks and Trials

Block	Discrimination task	Target for pressing key		Number of trials
		“E” key	“I” key	
1	Movie categorization	Movie^a^	No Movie^b^	20
2	Moral categorization	Dirty^c^	Clean^d^	20
3	Double congruent categorization	Movie/Dirty	No Movie/Clean	20 (practice) + 40 (test)
4	Reversed movie categorization	No Movie	Movie	40
5	Reversed double incongruent categorization	No Movie/Dirty	Movie/Clean	20 (practice) + 40 (test)

^a^One out of eight BDSM pictures from Fifty Shades of Grey. ^b^One out of eight mainstream pictures of weddings. ^c^One out of eight "dirty words": "Vomiting", "Diarrhea", "Sweat", "Spit", "Manure", "Mucus", "Anointed", "Sewer". ^d^One out of eight "clean words": "Laundry", "Soap", "Water", "Scented", "Snow", "Source", "Lily", "Shampoo".

The algorithm recommended by Greenwald et al. (2003) to calculate the D index has the following steps for IAT designs adopting the built-in error procedure: (1) use data from critical blocks (Blocks 3 and 5); (2) eliminate trials with latencies >10,000 ms; (3) eliminate subjects for whom more than 10% of trials have latencies <300 ms; (4) compute the standard deviation for all practice trials in the both congruent and incongruent blocks (Blocks 3 and 5) and the standard deviation for all test trials in both the congruent and incongruent blocks (Blocks 3 and 5); (5) compute separated means for practice congruent trials, practice incongruent trials, test congruent trials and test incongruent trials; (6) compute two difference scores (one difference between practice congruent trials and practice incongruent trials, and the other between test congruent trials and test incongruent trials); (7) divide each difference score by its associated standard deviation from Step (4); and (8) average the two quotients from Step (7), obtaining the D index.

A first sample 27 women were invited to take part in the experimental session but two of the women abandoned the session before completing the IAT task. Participants provided written informed consent to participate in the experiment. The retention interval between the movie’s release and the experiment was 39.12 days on average (*SD* = 5.49). The order of administration of the self-report explicit measures was randomized across participants, and the order of the administration of explicit and implicit measures was counterbalanced between subjects.

## Results

### Descriptive Analysis

To investigate the extreme emotions raised by the movie and the main erotic thread, descriptive analyses were run for each affective response and evaluation of the movie in the total sample (see Measures Section). Results showed that higher emotions felt were pleasure (*M* = 3.40, *SD* = 2.84), disappointment (*M* = 5.44, *SD* = 3.89), curiosity (*M* = 4.00, *SD* = 2.58) and reflections on one’s own romantic and sexual relations (respectively *M* = 3.48, *SD* = 3.72; *M* = 3.68, *SD* = 3.33). Additionally, participants showed medium-high levels of erotic, sadomasochistic, and perverse evaluation of the movie (respectively, *M* = 5.08, *SD* = 2.81; *M* = 4.84, *SD* = 2.85; *M* = 3.76, *SD* = 2.49).

### Differences in Sex Attitudes

Table 3 displays differences in sex attitude (erotophobic vs. erotophilic subsamples) for all measures considered in the study. An independent sample t-test was run on both explicit and implicit measures, with sex attitude (erotophobic vs. erotophilic) as a between-subjects factor. Given the limited sample size, and to prevent the violation of normal distribution assumptions, the non- parametric bootstrapping method was used as a robust estimation of t-test. Bootstrapping provided a confidence interval (CI) around the coefficients, which are significant if the interval between the upper limit (UL) and lower limit (LL) of a bootstrapped 95% CI do not contain zero, which means that the difference between the two groups is different from zero.

**Table 3:** Differences in Sex Attitudes (1000 Bootstrapped Samples)

Measure	Erotophobic (*n* = 13)		Erotophilic (*n* = 12)		*t*-test (*df* = 23)	95% Confidence Interval	Cohen’s *d*
	Min-Max	Mean (*SD*)	Min-Max	Mean (*SD*)			
Sexual Satisfaction Scale – Total	76-148	129.46 (20.86)	102-149	124.92 (15.73)	0.61	[-10.44, 18.08]	0.25
Sexual Satisfaction Scale – Contentment	16-30	24.69 (4.87)	13-30	21.25 (5.88)	1.60	[-0.70, 7.89]	0.67
Sexual Satisfaction Scale – Communication	13-30	23.46 (5.85)	14-30	23.83 (5.56)	-0.16	[-4.92, 4.20]	-0.07
Sexual Satisfaction Scale – Compatibility	13-30	24.77 (6.86)	10-30	22.83 (7.41)	0.68	[-3.77, 7.16]	0.28
Sexual Satisfaction Scale – Relational Concern	15-30	27.85 (4.28)	16-30	27.83 (4.06)	0.01	[-3.00, 3.28]	0.00
Sexual Satisfaction Scale – Personal Concern	14-30	28.69 (4.42)	24-30	29.17 (1.99)	-0.34	[-3.50, 1.72]	-0.14
Sex Guilt Scale – Total	7-31	20.54 (7.57)	0-19	9.08 (6.71)	3.99**	[6.03, 16.63]	1.67
Positive Emotions	0-7.75	2.83 (2.47)	0.50-6.50	3.56 (2.12)	-0.80	[-2.43, 1.16]	-0.33
Negative Emotions	0-5.50	3.50 (1.89)	0-6.75	2.88 (2.15)	0.77	[-1.00, 2.22]	0.32
Fantasies & Reflections	0-5.80	2.23 (1.95)	0.80-8.40	4.42 (2.59)	-2.40*	[-3.87, -0.42]	-1.00
Moral Evaluation^a^	0-7.33	1.64 (2.38)	0-3	0.25 (0.87)	1.91	[0.10, 2.78]	0.78
Sexual Evaluation	0-9	3.83 (2.53)	2-6.60	3.63 (1.55)	0.23	[-1.39, 1.74]	0.10
Romatic Evaluation	0-10	3.92 (3.17)	0-9.00	4.50 (3.48)	-0.43	[-3.14, 2.12]	-0.18
Sexual Motivation	0-9	1.64 (2.81)	0-7	1.53 (2.06)	0.11	[-1.73, 2.07]	0.05
Media Motivation	0-10	3.48 (3.02)	1-10	5.31 (2.99)	-1.51	[-4.05, 0.39]	-0.63
Mistake Motivation	0-10	2.00 (3.11)	0	0.00	2.23*	[0.50, 3.65]	0.93
*D*-index	-0.62-1.43	0.31 (0.56)	-0.57-1.10	0.18 (0.57)	0.57	[-0.35, 0.57]	0.24

*Note.* Min-Max from the empirical distribution.

^a^Bootstrapping was significant and effect size was high.

*p < .05. **p < .01.

No difference between the two groups in all scores of sexual satisfaction was found, confirming our idea that the difference investigated in our sample is only in terms of sexual attitudes, and not for sexual satisfaction or distress. Additionally, partially confirming Hypotheses 1 and 2, erotophobic women exhibited higher levels of sex guilt, fantasies and reflections after viewing the movie, and a more moral evaluation of Fifty Shades of Grey than erotophilic women. Furthermore, erotophobic women claimed to have seen the movie by mistake. No difference in terms of movie’s evaluation was found, meaning that for all participants the movie was seen as both romantic and erotic.

Confirming Hypothesis 3, all participants showed a positive average D value, suggesting an association between the Bondage-Domination-Sadism-Masochism pictures taken from the movie and dirtiness. No difference between the two groups in the D index was found, meaning that women who explicitly evaluated themselves as erotophilic and sexually emancipated showed a moralistic attitude towards sexual Bondage-Domination-Sadism-Masochism practices at an implicit level, just as erotophobic women do. According to Cohen (1977), the effect sizes can be considered high (see Table 3).

### Receiver-Operating-Characteristic (ROC) Analysis

To test Hypothesis 4, a receiver-operating-characteristic (ROC) analysis (Swets, 1988) was run to determine if and how well the IAT, at the implicit level, discriminated between erotophobic and erotophilic women at the explicit level. The D index was employed as a test variable indicating the "strength of conviction" that a participant falls into one category (erotophobic) or the other (erotophilic). An SOS score was employed as a state variable indicating the “true category” to which a subject belongs. The value of the state variable indicates which category should be considered positive (in our case erotophilic). ROC analysis provided an Area Under the Curve (A.U.C.), as a measure of discrimination which ranges from 1 (perfect discrimination) to 0 (null discrimination), with the value .50 representing random discrimination. The ROC analysis yielded an AUC of .45 when erotophilic was considered the positive value of the state variable. The ROC result suggested that, at the implicit level, the IAT did not discriminate women who explicitly evaluated themselves as erotophilic from ones who evaluated themselves as erotophobic. The IAT was not able to detect differences between erotophobic and erotophilic attitudes found at the explicit level. In other words, and confirming our expectations, explicit and implicit measures did not converge to discriminate women’s attitudes towards sexual morality.

## Discussion

E. L. James’s Fifty Shades of Grey has been the subject of a great deal of research (Comella, 2013; Downing, 2013; Stevens, 2014; van Reenen, 2014). Both the literary trilogy and the movie have created controversy rekindling the debate on consent and unusual sexual practices, such as BDSM. Generalizing over and above the specific context of the movie, sex may cause a variety of affective and cognitive responses and evaluations, such as positive affect, pleasure, satisfaction, a sense of freedom, but also fear of pleasure, shame, or guilt. A growing body of research has focused on explicit or self-reported attitudes towards sexuality (Fisher, Byrne, et al., 1988), while less is known about the role of implicit and automatic attitudes involved in sexual processing (Brauer et al., 2009; Oliver et al., 2009; Macapagal & Janssen, 2011).

The current pilot study aimed to explore women’s explicit and implicit attitudes towards an emancipated and liberal sexuality, such as that conveyed by the movie Fifty Shades of Grey. We aimed to explore the extent to which there is a difference in attitudes towards sexual morality on explicit and implicit levels. Results seemed to encourage our idea that erotophobic women explicitly exhibited higher levels of sex guilt and moral evaluation of the movie than erotophilic women, while the latter showed a higher level of fantasies and reflections than erotophobic women after seeing the move. Moreover, erotophobic women claimed to have seen the movie by mistake. On the contrary, no difference regarding implicit attitude was found in the two sexual opinion groups: All women showed a positive average value of the D index (Greenwald et al., 2003). In other words, all participants - both erotophobic and erotophilic - were faster in the IAT categorization when BDSM pictures taken from the movie were associated with dirty items (congruent block), than when the movie’s pictures were associated with clean items (incongruent block). This pilot study shows how explicit and implicit measures do not converge to discriminate women’s attitudes towards sexual morality: Also erotophilic women - who explicitly assessed themselves as not moralistic, not sexually guilty, and sexually emancipated, libertine, modern - associated the movie’s sexual perversions to the concept of dirtiness. Our findings display how explicit and implicit measures do not converge to discriminate women’s attitudes towards sexual morality.

A possible explanation of these results may be found in sex role stereotypes and socialization. In western society, for example, stereotypes for femininity include expectations that a woman be domestic, whole-hearted, pretty, emotional, dependent and passive. By contrast, masculinity stereotypes view them as unemotional, independent, active, and aggressive. Even if women are encouraged to appeal to stereotypical fantasies of heterosexual men when they show a true interest and pleasure in sex and unusual sex practices (especially outside the marriage), they are judged as being bad and immoral (Hamilton & Armstrong, 2009; Montemurro, 2014). As Simon and Gagnon (1986) noted, cultural settings are norms for sexual expression, such as the belief that women are sexually passive and men are sexually aggressive. As a consequence, we are prone to label as deviant women who initiate sex or men who are less interested in sex than their partners (Simon & Gagnon, 1986). In other words, we expect men to be more interested in sex and to engage in it more frequently than women because, many believe, that is how men are biologically wired (Montemurro, 2014).

Another possible explanation of our findings may be sought considering that Catholicism is deeply rooted in Italian culture due to the presence of the Vatican. As regards standards of sexual morality, according to the Catholic Church sexual pleasure is morally wrong when sought outside its procreative purposes (Catholic Church, 1997). As a consequence, for Catholics the release of the movie Fifty Shades of Grey denoted the evolution of pornography that is significantly distant from a sacred idea of sexuality and human dignity (Mohler, 2015). The previous record sales of the trilogy, and the actual celebration of the movie alerted many Catholics to the fact that a lost sense of shame is an increasing and unavoidable phenomenon (Mohler, 2015).

The facilitation and automatism of sorting the movie’s BDSM pictures portraying dirty concepts, as compared with the movie’s BDSM pictures showing clean concepts, reflect the presence of an implicit moralistic attitude towards some sexual practices such as bondage or sadomasochism, since that these sex activities are conceptualized as sinful, unclean, dirty, and morally guilty. Also women who explicitly evaluate themselves as erotophilic actually believe that sex is personally degrading and associated with base and animal instincts. The strong association between the movie’s pictures and dirty concepts, and between wedding pictures and clean concepts, was also confirmed by the positive average value of the D index in both erotophobic and erotophilic groups. The present findings might be explained by considering that when participants consider Bondage-Domination-Sadism-Masochism practices as guilty and dirty, the congruent categorization task appeared more facilitated, easier, and faster. The difficulty of switching between the two discrimination tasks (movie/no-movie pictures and dirty/clean words) when two concepts assigned to the same key are weakly associated (i.e. movie’s picture/clean word or wedding picture/dirty word) determines a slower performance (Nosek et al., 2007).

The main strength of the present work was its attempt to provide an innovative contribution to the investigation of explicit and implicit attitudes in the sexual domain. The present study has however some theoretical and methodological limitations. First, our sample size was relatively small. Second, our female participants were not selected on the basis of highest erotophobia– erotophilia scores, but instead they were assigned to two groups on the basis of a median SOS score. Lastly, future studies could recruit a larger sample including male participants, and also assess the participants’ level of religious attitudes, involvement, and affiliation. However, despite the sample size, effect sizes were large throughout analyses, indicating that these findings represent meaningful results. Although a pilot version, we tried to demonstrate that group differences in erotophobia – erotophilia were not related to implicit attitudes towards unusual sex practices and that women are not completely aware or are hesitant or reluctant to sustain their sexual emancipation for fear of being morally judged. Despite the above cited limits, the present study is an attempt to address the issue of explicit and implicit attitudes towards unusual sex activities, and adds to our knowledge about the key role that moral culture and religion still play in the sexual domain.
